# Preoperative CT-based 3D analysis of middle colic vein anatomy for complete mesocolic excision in right-sided colon cancer: a retrospective cohort study from a prospective clinical trial

**DOI:** 10.1007/s00276-026-03987-8

**Published:** 2026-09-02

**Authors:** Vladimir Zivanovic, Dejan Ignjatovic, Javier Armando Luzon, Bojan Vladimir Stimec

**Affiliations:** 1 University Hospital Centre “Dr Dragisa Misovic–Dedinje”, Belgrade, Serbia; 2 Department of General Surgery with Anesthesiology, Faculty of Medicine, University of Belgrade, Belgrade, Serbia; 3 Department of Digestive Surgery, Akershus University Hospital, Nordbyhagen, Norway; 4 Institute of Clinical Medicine, Faculty of Medicine, University of Oslo, Oslo, Norway; 5 Department of Diagnostic Imaging, Akershus University Hospital, Nordbyhagen, Norway; 6 Anatomy Unit, Faculty of Medicine, University of Geneva, Rue Michel-Servet 1, Genève 4, 1211 Switzerland

**Keywords:** Alternative confluence pattern, Anatomic variations, Cancer, Colon, 3D CT analysis, Lymphadenectomy, Middle colic vein (MCV)

## Abstract

**Purpose:**

Complete mesocolic excision requires central mesenteric dissection near the middle colic vein (MCV) confluence, where anatomic variability may increase operative risk. However, MCV multiplicity, confluence patterns, and relationships to the middle colic artery (MCA) are not sufficiently defined.

**Methods:**

We performed a retrospective secondary analysis of 300 consecutive three-dimensional vascular reconstructions from a prospectively maintained database of patients undergoing surgery for right-sided colon cancer (age 65 ± 9 years; 59% female) between November 2017 and December 2022. We classified transverse mesocolic venous trunks operationally as MCV1, MCV2, and MCV3 according to their right-to-left position to standardize the description of multiple venous drainage patterns. MCV trunks (MCV1–3), their confluence patterns, and their spatial relationships to the MCA were analyzed using descriptive statistics. The reconstruction method had been previously validated intraoperatively and was performed by a single investigator.

**Results:**

The MCV was identified in all cases (397 veins total). There was one trunk in 68.7%, two in 30.3%, and three in 1.0% of patients. Confluences were most often into the superior mesenteric vein (65.7%), followed by the inferior mesenteric vein (12.6%) and gastrocolic trunk (9.8%). MCV1 drained mainly into the SMV (72.7%). MCV2 showed variable drainage into the SMV (45.7%), IMV (31.9%), jejunal vein (9.6%), left SMV (3.2%), and left colic angular vein (2.1%). MCV3 drained into the IMV in 66.7% and the left SMV in 33.3%. The MCV and MCA were separate in 67.0% of cases, with anterior crossing in 17.7% and posterior crossing in 15.3%. Left-sided drainage patterns were seen in 20–50% of patients with 2 trunks and 3.

**Conclusions:**

MCV anatomy is highly variable, with multiplicity in 31.3% and frequent alternative confluence pattern. Preoperative 3DCT can identify alternative variants and may support safer, tailored vascular ligation during lymphadenectomy.

**Supplementary Information:**

The online version contains supplementary material available at https://doi.org/10.1007/s00276-026-03987-8.

## Introduction

The development of oncologic surgery over the last few decades has introduced broader surgical dissection to achieve adequate lymph node harvest [23]. Thus, complete mesocolic excision (CME) with central vascular ligation and D3 lymph node dissection has led surgeons to dissect the central mesentery in the area of the pancreatic notch, namely, the area of the superior mesenteric vessels, the Middle Colic Artery (MCA) origin, and Middle Colic Vein (MCV) confluence [16, 14]. Of all these vessels, the MCV has received the least attention [5]. While CME with D3 central vascular ligation was proposed as a more radical oncologic approach, large Japanese and European series have shown excellent outcomes after conventional right colectomy with D2 lymphadenectomy, suggesting that extended D3 dissection should be used selectively rather than routinely, particularly for right‑sided colon cancer [23].

In our earlier research, we demonstrated a 6.7% positivity rate for lymph nodes in the D3 field, which consequently necessitates a Right colectomy with D3 lymphadenectomy [7].

The middle colic vessels represent one of two principal vascular pedicles encountered during right-sided colon resection and present critical anatomical landmarks in CME. Their systematic identification, central ligation, and *en bloc* resection are fundamental for achieving an adequate oncologic specimen, ensuring proper lymphovascular clearance. The MCV serves as the primary drainage vessel for the transverse colon, typically emptying into the SMV, alongside the trajectory of the MCA as it traverses the mesocolon transversum [33]. Subsequently, blood is led into the hepatic portal system through the portal vein. The veins and their typical drainage points are important in colorectal surgery because injuries or variations can affect surgical outcomes and present as bleeding risks [33]. While the number of MCVs and the positions of their confluences have been reported in the literature, the confluences of the second/third MCV, as well as the confluence pattern and the topographic relations to the MCA trunk still remain unreported [22].

Preoperative workup for colon cancer surgery includes computerized tomography (CT) imaging for disease staging [4]. The CT dataset also offers crucial preoperative insights on the vascular anatomy of the specific patient [5]. The radiologist and surgeon can study the staging CT or be provided with a CT-based 3D vascular reconstruction, that will facilitate both the reporting of variations by radiologists and intraoperative orientation for surgeons [38, 24]. In this manner, the surgeon can evade pitfalls and shorten operative time when performing CME [27].

This study aims were to characterize venous anatomical variants and their relationship to central arterial trunks in patients undergoing D3 right colectomy, using 3D MDCT angiography and validated segmentation. Operative and long‑term oncologic outcomes of D3 right hemicolectomy have been reported in separate clinical studies and are beyond the scope of the present anatomical analysis. The aim of this study was also to analyze the number of MCVs, their confluences in total and according to their position (MCV1, 2, and 3). A further aim was to compare the confluence pattern of the MCV2 and 3 to that of the MCV1. The third aim was to analyze and categorize the topographic relations found between the MCV and the MCA trunk.

## Materials and methods

### The CT datasets

A Retrospective Cohort Study from a Prospective Clinical Trial “Safe Radical D3 Right Hemicolectomy for Cancer through Preoperative Biphasic MDCT Angiography.” A total of 300 MDCT datasets from patients enrolled in that trial were available for evaluation. The parent study was registered at ClinicalTrials.gov (NCT01351714) and approved by the Norwegian South-East Regional Ethical Committee (2010/3354). All participants signed informed consent forms for inclusion in the study and the use of the collected data in future publications. This included the uploading of the preoperative CT dataset through a safe FTP server in the Anatomy Unit, Faculty of Medicine, University of Geneva, Switzerland, where these were reconstructed in 3D and thereafter returned to the operating surgeon.

Inclusion criteria were: (1) histologically confirmed right-sided colon cancer; (2) operative treatment with right colectomy / D3 lymphadenectomy; and (3) available contrast-enhanced MDCT angiography with successful three-dimensional vascular reconstruction. There were no exclusions since all CT datasets were successfully reconstructed. Only vessels in the central mesentery were reconstructed and used to navigate the lymphadenectomy during the operative procedure. The selection of the 300 most recent 3D reconstructions was made to ensure consistent quality of datasets, namely ≤ 1 mm slice interval, as it provides superior reconstructions.

### Image acquisition and processing

The multidetector computed tomography angiography examinations were performed with a 64- detector CT scanner (Siemens, Erlangen, Germany). Portal-venous phase images were obtained after contrast media administration (Iohexol 350 mg/ml, Omnipaque 350 mg I/mL; GE Healthcare) using a power injector at a rate of 3–5 ml/sec followed by a 30-ml saline chaser. Scan delay for acquisition of portal phase images was obtained by means of bolus tracking positioning. Scan pitch was 1.375, tube voltage was 120 kVp, and automatic tube current modulation was used with a maximum effective tube current–time product of 400 mAs. The dataset images were reconstructed with a 0.67–1.00 mm section thickness and a 0.452 mm reconstruction interval.

The DICOM datasets underwent minute image analysis through manual segmentation and 3D reconstruction, presenting the complete mesenteric vascular tree – the superior mesenteric vessels and their branches and affluents, up to at least 4th order of branching. The datasets were initially reconstructed in 3D using the FDA-approved software Osirix MD v.14.0 (Pixmeo, Bernex, Switzerland), which is available for the Macintosh platform. Subsequently, for the final validation, Mimics Medical imaging processing software, version 24.0, along with the 3-matic medical software version 16.0, were employed on a computer running Windows 10 Pro x64 2017 (Materialise NV, Leuven, Belgium). The manual segmentation in Osirix was obtained by serial application of Region of Interest (ROI) through editing tools: Open polygon, Pencil, and Repulsor on each slice. After creating this series of ROIs, the pixel values outside them were validated to air (-1’024), thus erasing the surrounding elements, and the interior of the ROIs was set to their original value. The virtual model was obtained by 3D volume rendering (VR), including still images, videos, and stereolithography (STL) files. The 3DVR models underwent morphometry with the aid of the Length tool. Likewise, the manual segmentation in Mimics was obtained via Profile line thresholding, Single and Multiple slabs editing with interpolation, Dynamic region growing, 3D Interpolate, and Boolean operations. The final product of the imaging was a 3D model. The morphometry of the vascular elements (in centimeters) in the 3DVR file was carried out through 3-matic medical, using the Radius (for calibers) and Length (over the surface, true shortest path) tools. Each measurement was performed twice, and the mean value was accepted as final. The 3D virtual representation of the vascular tree after this comprehensive morphological and morphometric analysis was ultimately exported as STL files or 3D PDFs with annotations. The 3D models enabled deep zooming in and rotation in all planes and subsequently precise identification of venous trajectories and their confluence points. Also, this manipulation clearly avoids potential confusion of vessels’ overlapping in 2D. The segmentation method and morphometry have been performed by a highly competent and experienced clinical anatomist (BVS) who has over 1000 cases of successful manual segmentations of abdominal CT datasets in the context of clinical studies, all validated at surgery and published [36, 28]. The manual segmentations have demonstrated superiority over semiautomatic segmentation [20]. We examined the venous blood vessels within the region of interest (ROI) and conducted identification, determining the caliber where feasible and the confluence position of the specified venous vessel [3]. The MCV is recognized as the main venous structure draining blood from the transverse colon. Particular emphasis was placed on the reconstruction of the following veins: the right colic (RCV), ileocolic (ICV), gastrocolic trunk of Henle GTH, LCAV, pancreaticoduodenal vein (PDV), terminal ileal venous trunk (TIVT), right colic (RCV), right gastro-omental vein (RGOV), IMV, jejunal (JV), as well as bifid SMV with right (RSMV) and left (LSMV) trunks, when present.

### Anatomical nomenclature

For this study, the term MCV was used as an operational definition for the venous trunk(s) draining the transverse colon within the D3/right mesocolic region. When more than one such trunk was present, they were designated from right to left of the patient as MCV1, MCV2, and MCV3. These labels were applied consistently to facilitate standardized anatomical reporting and do not imply that only one “main” venous trunk must exist. Preoperative 3D CT angiography was a requirement and was used as a navigation tool at surgery. The protocol does not allow this surgery without the vascular map.

### Operative procedure

Right colectomy with CME and D3 lymphadenectomy was performed following standardized institutional protocols, with medial‑to‑lateral dissection, central ligation of ileocolic and right colic vessels (when present), and *en bloc* removal of a personalized D3 mesenteric volume, as previously defined by our research group [32]. Procedures were carried out through open or laparoscopic approaches; while robotic access was introduced later and was not analyzed separately in this anatomical study.

In summary, three-dimensional (3D) images reconstructed preoperatively from staging CT scans serve as a surgical roadmap. A transverse incision is made in the visceral peritoneum over the terminal ileal vessels, positioned 1 cm caudal to the ileocolic artery’s origin. Once the SMA and SMV are identified and their vascular sheaths opened, the terminal ileal vessels are secured using vessel loops. The anterior flap of mesenteric tissue along the left side of the SMA is developed, and the surgical specimen is de-vascularized by dividing the ileocolic artery (ICA), right colic artery (RCA), and MCA or its right branch at their origins. The surgical specimen is fully mobilized to allow access to the posterior flap of mesenteric tissue. The medial border of this posterior flap, located behind the SMV and SMA, is divided along a line parallel to the left border of the SMA, extending from the ICA’s origin to the MCA’s origin. This is done after gently “rolling” the SMV to the left and dividing its vascular sheath along with that of the SMA, effectively removing complete arterial stumps. Consequently, all tissue, both anterior and posterior to the mesenteric vessels, is excised *en bloc* with the specimen that includes the central lymph nodes and vessels, referred to as the D3 volume. All surgical specimens are then sectioned along a line that is parallel to and 1 cm lateral from the right side of the SMV in order to separately examine the D3 volume for lymph node metastasis [13].

### Statistics

For continuous variables, the mean ± standard deviation was utilized, while categorical values were presented as numbers (percentages of the total repetitions for each category). Excel for Mac ver. 16.103.4 software was used for the mean and standard deviation.

## Results

Three hundred most recent, consecutive 3D vascular reconstructions of patients who underwent surgery for right-sided colon cancer were analyzed (mean age 65 ± 9 years, 178 (59.33%) female). The surgeries were performed from November 2017 until December 2022.

1. MCVs and their confluences.

The MCV was present in all patients (300/100%). Overall, we detected a total of 397 MCVs. There was one MCV trunk in 206 patients (68.7%), 2 trunks occurred in 91 patients (30.3%), while a situation with 3 MCV trunks occurred in 3 patients (1%). A MCV2 was found in 94 patients (31.3%), while MCV3 occurred in only 3 patients (1%).

Confluences were analyzed for all 397 MCVs in 300 patients. The majority of MCVs had their confluence to the SMV (65.7%) for the whole group, while the second most frequent confluence position was unexpectedly the IMV. All these IMVs also terminated into the SMV. The variability of MCV confluences is presented in Table 1. Moreover, a subgroup consisting of 11 double SMVs (right SMV and left SMV) was encountered. In 7 of these cases (single MCV), the MCV followed the usual trajectory terminating in the RSMV trunk. A further 3 cases with two MCVs had a confluence to both SMV trunks, the MCV1 to the rSMV and MCV2 to the lSMV trunk. A single case with a three MCVs revealed the MCV1 and 2 draining into the rSMV, and the MCV3 to the lSMV trunk.

**Table 1:** Confluences of MCV into the collecting venous blood vessel

MCV1	SMV	IMV	GTH	JV	SV	LSMV	LCAV	∑
	218	18	38	17	8		1	300
MCV2	43	30	1	9	6	3	2	94
MCV3		2				1		3
∑	261	50	39	26	14	4	3	397

MCV − middle colic vein, SMV – superior mesenteric vein, IMV – inferior mesenteric vein, GTH – gastro colic trunk Henley, JV – jejunal vein, SV – splenic vein, LSMV – left superior mesenteric vein, LCAV – left colic angle vein

2. Stratification of confluences and trajectories for MCV1, 2, and 3.

Further stratification of the MCV confluences was based on the number of MCVs per patient, namely one (MCV1), two (MCV2), and three (MCV3). Table 1 demonstrates that the MCV1 tends to follow a normal trajectory in 72.7% of patients, while the MCV2 and MCV3 tend to follow an alternative trajectory.

**Fig. 1:**
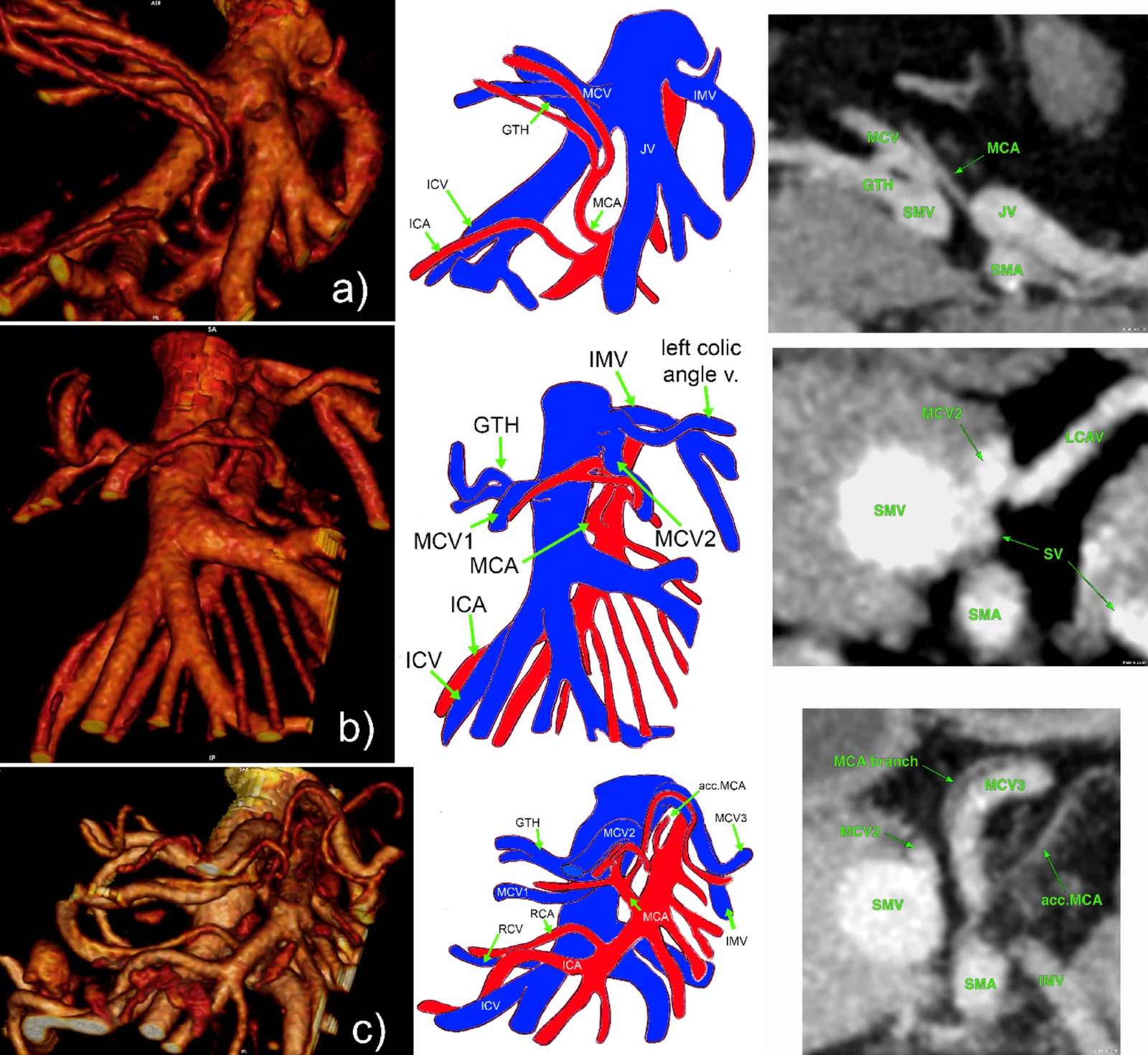
Cases with single, double, and triple MCV. Osirix 3DCT reconstruction on the left and Multiplanar reconstruction - MPR on the right. **a** MCV drain into SMV; **b** MCV1 drain into GTH and MCV2 drain into SMV; **c** MCV1 and MCV2 into SMV, MCV3 into IMV. MCV - middle colic vein, SMV – superior mesenteric vein, IMV – inferior mesenteric vein, MCA – middle colic artery, GTH – gastrocolic trunk Henle, JV – jejunal vein, ICV – ileocolic vein, ICA – ileocolic artery, TIVT - terminal ileal venous trunk, RCA-right colic artery, SMA – superior mesenteric artery, LCAV – left colic angle vein, SV – splenic vein, RCV – right colic vein, acc MCA – accessory middle colic artery

**Fig. 2:**
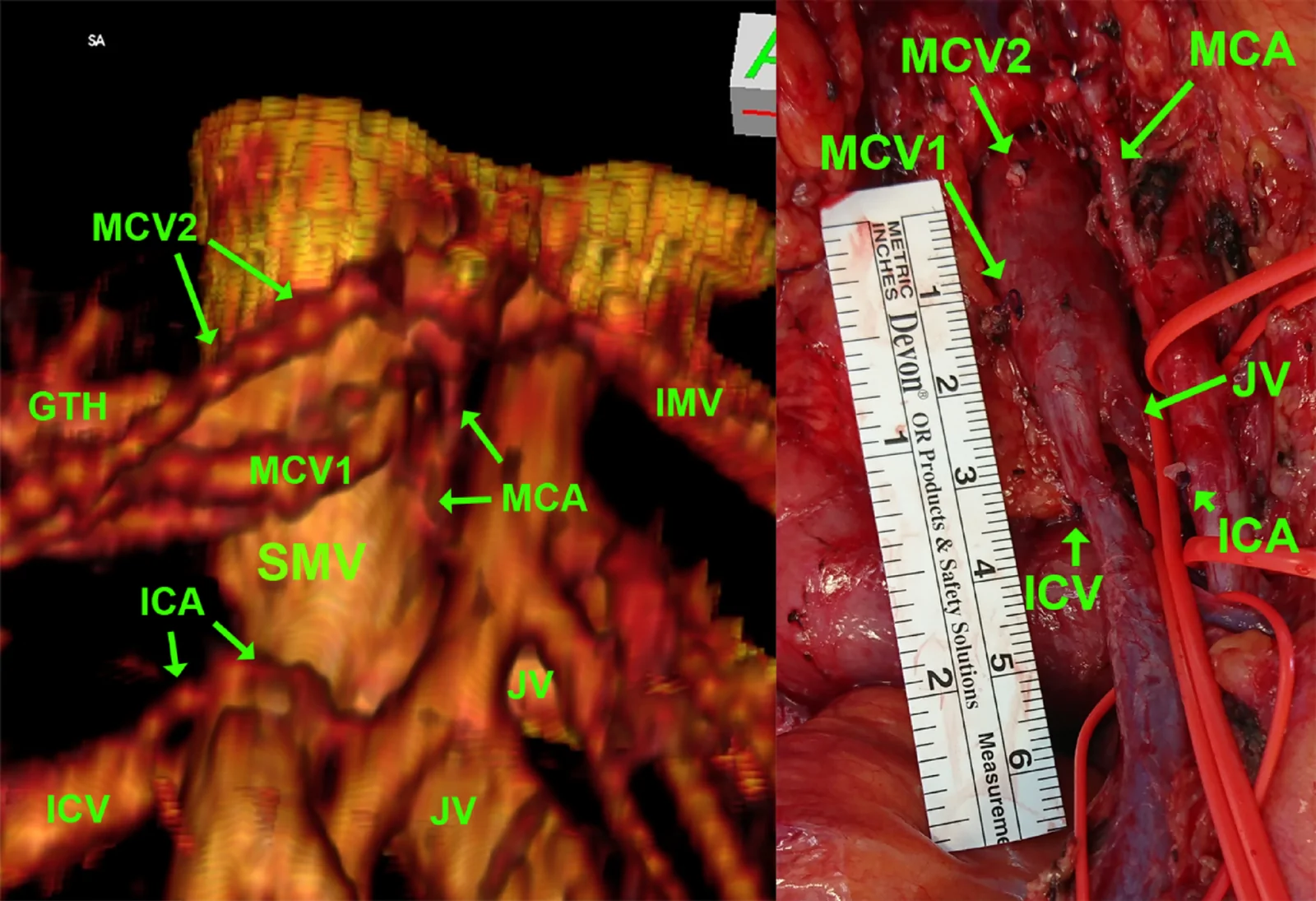
Osirix 3DCT reconstruction and picture at the open surgery: MCV 1,2 entering superior mesenteric vein. MCV - middle colic vein, IMV – inferior mesenteric vein, SMV – superior mesenteric vein, MCA – middle colic artery, GTH – gastrocolic trunk Henle, JV – jejunal vein, ICV – ileocolic vein, ICA – ileocolic artery

**Fig. 3:**
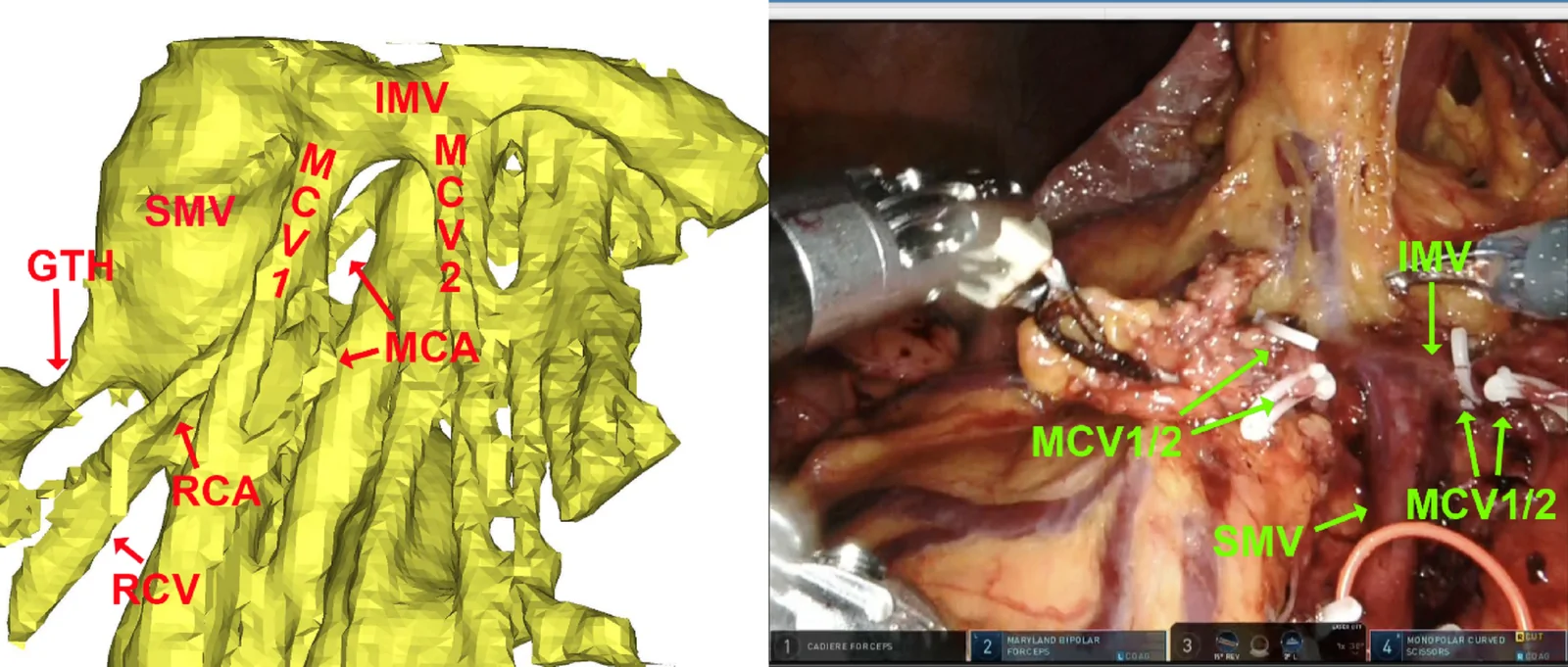
Materialise Mimics 3DCT reconstruction and picture at the robotic surgery: MCV 1,2 entering inferior mesenteric vein. MCV - middle colic vein, IMV – inferior mesenteric vein, SMV – superior mesenteric vein, MCA – middle colic artery, GTH – gastrocolic trunk Henle, RCA-right colic artery, RCV – right colic vein

**Fig. 4:**
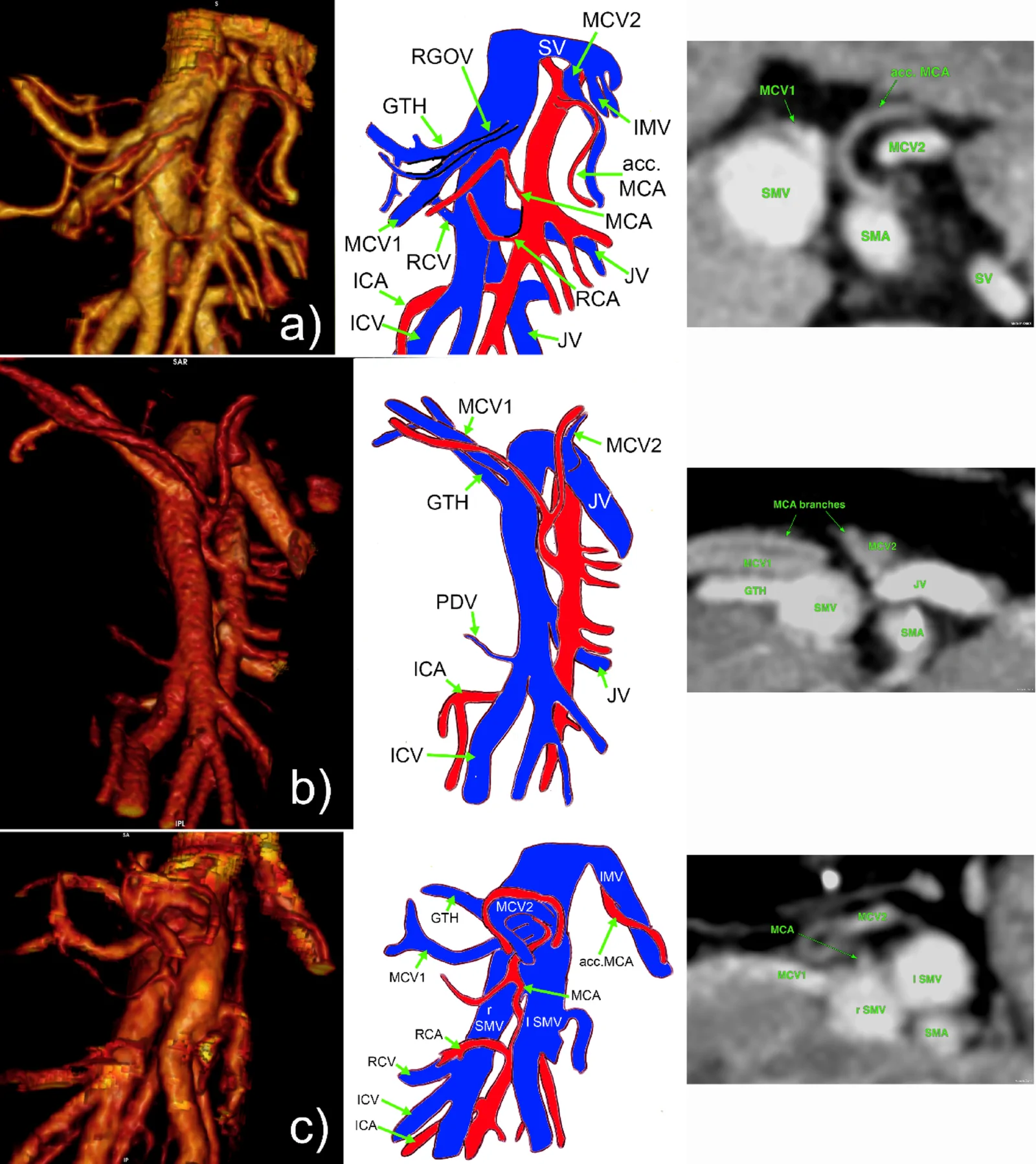
Cases with aberrant confluences. Osirix 3DCT reconstruction on the left and Multiplanar reconstruction - MPR on the right. **a** MCV2 drain into SV; **b** MCV2 drain into JV; **c** MCV2 drain into lSMV. MCV - middle colic vein, SMV – superior mesenteric vein, IMV – inferior mesenteric vein, lSMV-left superior mesenteric vein, rSMV-right superior mesenteric vein, MCA – middle colic artery, GTH – gastrocolic trunk Henle, JV – jejunal vein, ICV – ileocolic vein, ICA – ileocolic artery, TIVT - terminal ileal venous trunk, RCA-right colic artery, RCV – right colic vein, RGOV – right gastroomental vein, SV – splenic vein, acc MCA – accessory middle colic artery, PDV – pancreatico duodenal vein

**Fig. 5:**
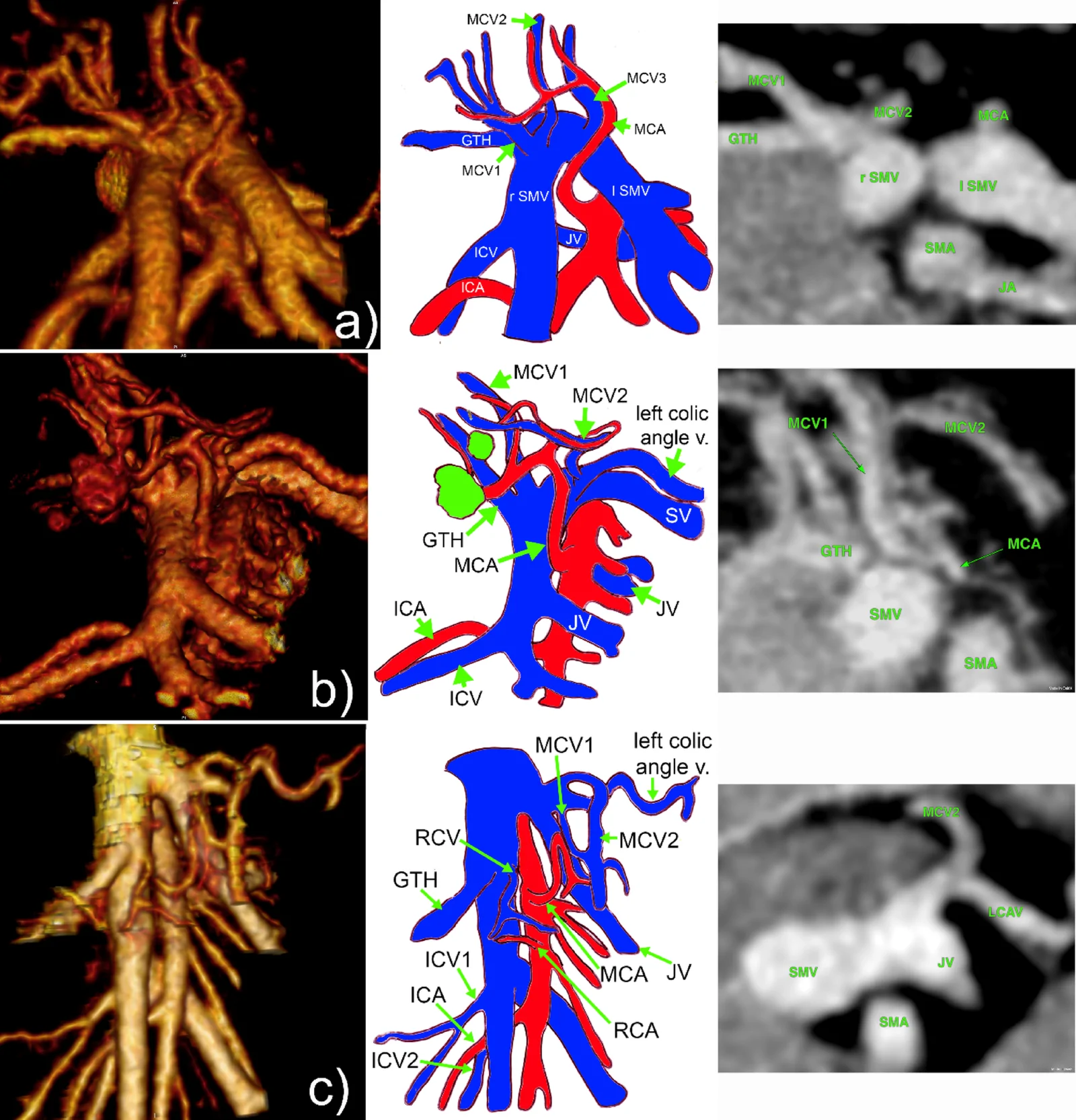
MCV Trajectories. Osirix 3DCT reconstruction on the left and Multiplanar reconstruction - MPR on the right. **a** MCV1 drains side-by-side with the GTH to the RSMV. MCV2 drains only 0.28 cm to the left of MCV1 base, also to the RSMV. MCV3 drains to the terminal portion of the lSMV. All three MCVs are followed by MCA branches, which pass inferiorly, i.e., superficial to them; **b** MCV1 drains into the SMV side-by-side with the GTH. MCV2 drains into the terminal portion of the LCAV, just before it terminates in the proximity of the splenomesenteric junction. The MCV is followed by the MCA left branch, which runs first on its deep (upper surface), then on its left-hand side. The MCV2 is directed to the right, entering the drainage area of the MCV1 and GTH; **c** MCV1 and MCV2 drain into the terminal portion of the LCAV, just before this vein itself terminates in the LSMV. MCV1 drains from below, and the MCV2 from above. However, both MCVs descend in front of the lSMV and the LCAV and anastomose within the transverse mesocolon in the form of a precoce arcade. They are followed by the right branch of the MCA, while the left MCA branch is concomitant to the LCAV. MCV - middle colic vein, SMA – superior mesenteric artery, SMV – superior mesenteric vein, IMV – inferior mesenteric vein, lSMV-left superior mesenteric vein, rSMV-right superior mesenteric vein, MCA – middle colic artery, GTH – gastrocolic trunk Henle, JV – jejunal vein, JA – jejunal artery, ICV – ileocolic vein, ICA – ileocolic artery, TIVT - terminal ileal venous trunk, RCA-right colic artery, LCAV – left colic angle vein, SV – splenic vein

- An MCV1 confluence to the SMV was present in 218 (72.7%), the GTH in 38 (12.7%), the IMV in 18 (6.0%), the JV in 17 (5.7%), the SV in 8 (2.7%) and LCAV in 1 (0.3%) (Fig. 1a).
- For the whole group with two MCVs (94 cases) the MCV2 confluence was to the SMV in 43 (45.7%), IMV 30 (31.9%), JV 9 (9.6%), SV 6 (6.4%), LSMV 3 (3.2%), LCAV 2 (2.1%) (Fig. 5b, c), and to the GTH 1 (1.1%) cases (Table 1. Figure 1b). Out of the 94 cases with at least two MCVs, 47 (50.0%) were cases where MCV1 and MCV2 terminated into the same vessel, most often to the SMV (39, 83%) (Figs. 2 and 3). In the remaining half (50.0%), they terminated into different vessels. In this group, the trajectory of the MCV1 was to the patient’s right, while the trajectory of MCV2 was always to the left (Fig. 4a, b, c).
- When three MCVs are in concern, the MCV1 and MCV2 trajectories are to the patient’s right, while that of the MCV3 is to the left, namely, the confluences of the MCV3 were to the IMV in 2 cases (66.6%) (Fig. 1c), and to the lSMV in 1 (33.3%) case (Fig. 5a).

3. Relationship between MCV and MCA trunk.

**Fig. 6:**
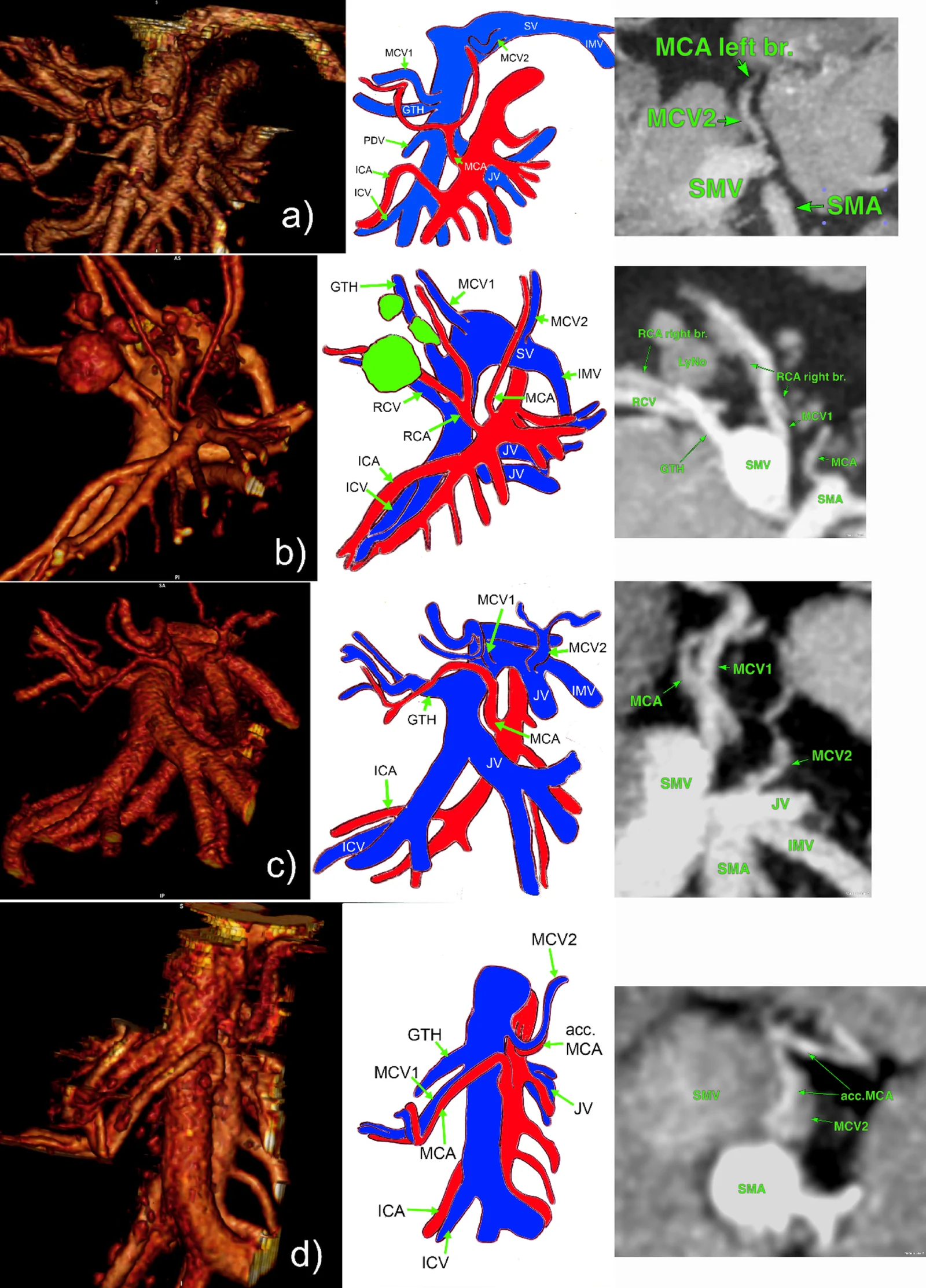
Topography of MCV. Osirix 3DCT reconstruction on the left and Multiplanar reconstruction - MPR on the right. **a** MCV and MCA were separate and did not intersect; **b** MCV in front of MCA; **c** MCV and MCA were separate but close. Confluence of JV, MCV2, and IMV; **d** MCA in front of MCV. MCV - middle colic vein, IMV – inferior mesenteric vein, PDV – pancreatico duodenal vein, SV – splenic vein, MCA – middle colic artery, GTH – gastrocolic trunk Henle, JV – jejunal vein, ICV – ileocolic vein, ICA – ileocolic artery, RCA-right colic artery, SMA – superior mesenteric artery, SMV – superior mesenteric vein, JV – jejunal vein, acc MCA – accessory middle colic artery

In 201 instances (67%), the MCV and MCA were separate and did not intersect (Fig. 6a, c). In 53 instances (17.7%), the MCV crossed in front of the MCA (Fig. 6b), while in 46 instances (15.3%), the MCA crossed in front of the MCV (Fig. 6d). There were no instances of topographic relations of the MCA trunk with more than one MCV.

4. The jejunal veins (JV).

JV occurred in 291 (97%) patients, resulting in a total of 593 JV. The remaining 9 (3.0 %) were cases of double SMVs. Overall, 11 cases with a double SMV were found. Nine of these cases didn’t have JVs, namely, the LSMV trunk replaced the JVs (Fig. 4c). In 2 cases with a double SMV, a JV crossed the SMA posteriorly and drained into the RSMV (Fig. 5a).

**Fig. 7:**
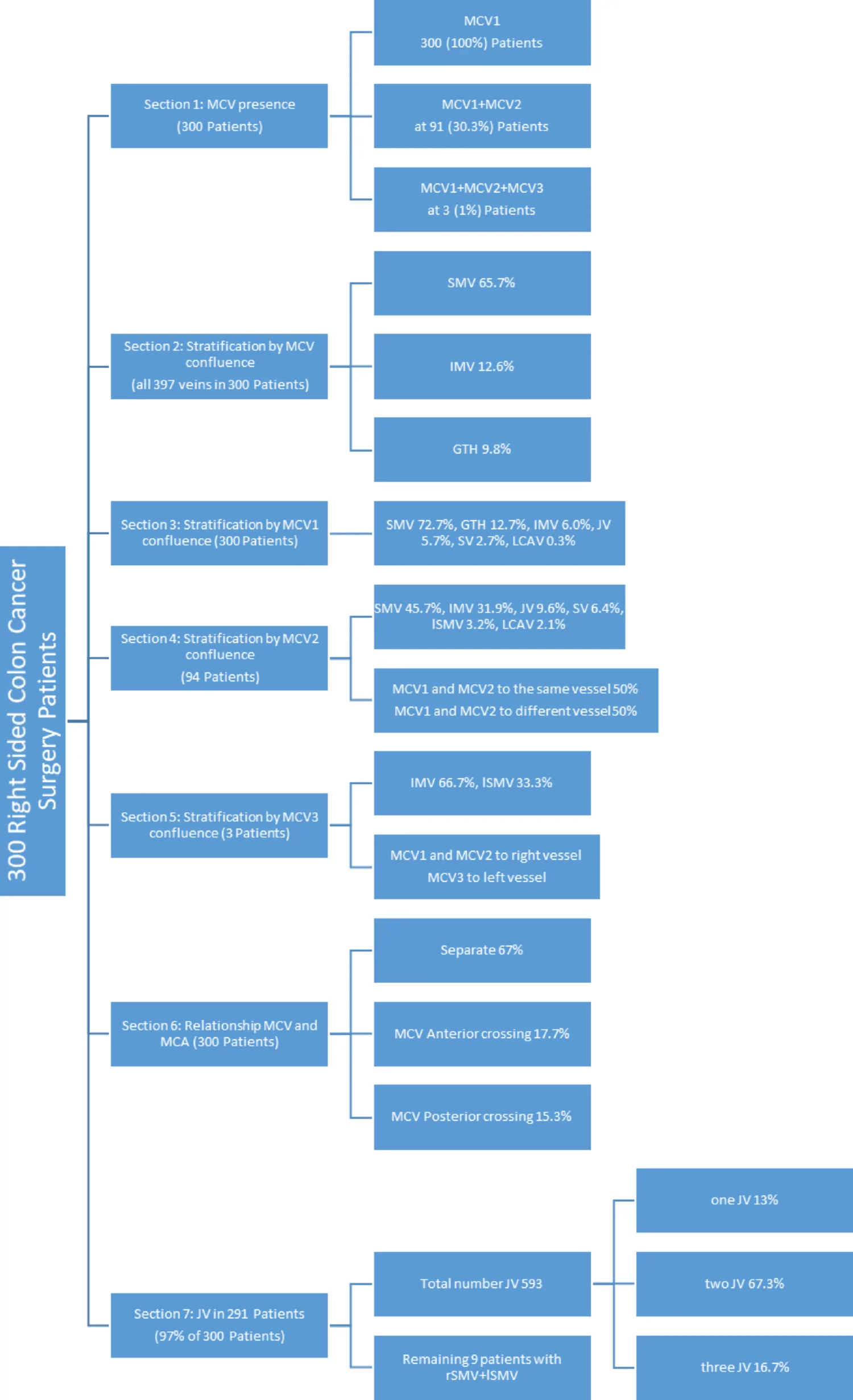
Flow chart with MCVs confluence and JV presence. MCV - middle colic vein, IMV – inferior mesenteric vein, SV – splenic vein, GTH – gastrocolic trunk Henle, JV – jejunal vein, LCAV – left colic angle vein, lSMV - left superior mesenteric vein, rSMV - right superior mesenteric vein, RCA-right colic artery, SMV – superior mesenteric vein, JV – jejunal vein

A single JV was found in 39 (13%) patients, with an average caliber of 0.85 ± 0.26 cm, while two jejunal veins were present in 202 (67.3%) patients. The average caliber of the second JV measured 0.71 ± 0.24 cm, and in 50 (16.7%) patients, where a third JV was found, the average caliber was 0.61 ± 0.22 cm. (Fig. 7)

5. Additional anatomical structures and measurements of interest:

We present the venous vessels of interest in the order of occurrence: TIVT (300, 100%), ICV (298, 99.3%), GTH (289, 96.3%), LCAV (70, 23.3%), PDV (68, 22.7%), RCV (59, 19.7%), and the RGOV (34, 11.3%) patients. The mean distance from ICV to GTH measures 3.51 ± 1.02 cm, and ICA to MCA measures 2.24 ± 1.13 cm.

## Discussion

The main finding of this study was the overwhelming and unexpected number of MCV variations when venous multiplicity and confluence variability are combined with the topographic variety of the MCVs described in this article. The consistency of the MCV, namely, its presence in all patients (100%), does not imply simple anatomical relations.

The Literature states that most commonly, a single middle colic vein occurs in approximately 72–84% of individuals [40]. Two middle colic veins are found in 10–46% of cases [30]. Three middle colic veins are less common, occurring in 1–12% of individuals [1]. When compared with our results, we have a slightly lower proportion of cases with one MCV (68.7%), while the patients with at least two MCVs (30.3%) are consistent with the literature. A triple MCV was found only in 1.0% of our material, falling low in the above-mentioned range. Maki et al. have stated that [8] the thickest, dominant MCV is considered the “main” MCV because it is, as the authors claim, clinically most important during surgery, due to the risk of bleeding [39, 6]. In our experience, we have had the impression that the MCV1 and MCV2 were of similar caliber and thicker than that of the MCV3; thus, we named the veins as MCV 1, 2, and 3, going from the patient’s right to the left.

Among the 300 MCV1 vessels, 218 (72.7%) drained directly into the SMV, somewhat higher than reported by Maki et al. (62.5%) [22]. These authors, however, show a much higher GTH confluence rate (29.3%) vs. 12.7%, implying a possibly different pattern of variations in different populations [12]. The same can be said for the less common sites that include the IMV at 6% vs. 4.8%, SV at 2.7% vs. 2.7%, and a considerably more frequent (10-fold) confluence rate to the JV in our series at 5.7 vs. 0.6% [22, 15]. To our knowledge, a confluence on the LCAV at 0.3% has not been described in the literature. Given that our reconstruction technique, the thickness of the CT slices, and the sample size align closely with the study conducted by Maki et al., the significance of this comparison is greatly improved.

Although some venous variants — particularly MCVs draining into the IMV—may theoretically increase the risk of central venous injury, our retrospective anatomical design did not allow robust correlation of individual patterns with intraoperative bleeding or complications. Prospective studies such as those by Willard et al., combining standardized preoperative 3D mapping with detailed operative complication reporting, are warranted to clarify the clinical impact of these variants [38].

When compared to the MCV1, the deviation of the MCV2 termination from common anatomy seems to be more pronounced. Namely, the SMV remains the most common confluence site; however, there is a notable decline to 45.7%, while the IMV rises to second place at 31.9%. The JV follows with 9.6%, after that the SV with 6.4%, the LSMV with 3.2% and the LCAV confluence with 2.1%. Surprisingly, the rarest confluence of the MCV2 was to the GTH, encountered in only one 3D reconstruction [19]. Another curiosity, not previously reported in the literature, is LSMV confluence that was found in 3 cases in our series. Further analysis of cases involving the three MCVs reveals an even higher variability in confluence patterns. MCV3 were found in 3 cases, draining to the IMV (2 cases, 66.7%) and to a pseudo bifid lSMV trunk (1 case, 33.3%), with no SMV or GTH drainage noted [25, 34]. Multiple MCVs, thus, favor aberrant paths, emphasizing the anatomical complexity that surgeons face, and showing an increased left-sided or atypical drainage in 20–50% of multi-vein cases [26]. Nevertheless, in all cases, all confluences were to the veins of the portal circulation [21].

The narrow area of dissection within the pancreatic notch represents a challenge for the operating surgeon [17]. Bleeding can arise from unforeseen locations, as indicated by the venous confluences (SMV, IMV, JV, LCAV, GTH) in case of MCV injury. Performing a lymphadenectomy (in right colectomy) may represent a greater challenge when the MCV2 and/or MCV3 are present.

These anatomical patterns may be useful for generating hypotheses about individualized dissection strategies in future studies, which will be based on the surgeon’s awareness of the anatomy before surgery. The fact that the MCV1 most often terminates into the SMV (72,7%), the MCV2 considerably less often (45,7%), and the MCV3 never (0%) can imply that MCV2 and MCV3s not draining into the SMV basin can be preserved when performing right colectomy, in this manner not enlarging the dissection area. In theory, when part of the transverse colon is preserved, and multiple MCVs are present, selective ligation of the first draining vein (MCV1) with preservation of other veins (MCV2/MCV3) might help maintain venous outflow. In standard radical right colectomy with transverse colon resection, all major draining veins must be controlled, and oncologic and technical safety override any attempt at venous preservation.

The remaining factor contributing to the variety of the MCV is its topographic relationship to the MCA trunk [29]. Due to the size of the superior mesenteric vessels as well as the levels of the MCV confluences and the MCA origins, a substantial gap exists between these two structures [35]. Thus, it is no surprise that “no topographic relation” is found in 67.0% of cases. However, clinical value can be assigned to the two remaining variants (MCV or MCA lies anterior or posterior), which comprise 33.0%. Recognizing these variants before surgery could be crucial, as additional caution is required when dissecting the vein first, since it is more susceptible to traction and accidental damage, as troublesome bleeding can occur [2, 8, 18]. This variant occurs in 17.7% of cases, while the posterior variant occurs in 15.3% and could be just as dangerous.

Intraoperative bleeding from the middle colic vein or its tributaries—specifically during the dissection (traction and countertraction) of the gastrocolic trunk of Henle (GCTH) — is a recognized complication during laparoscopic right colectomy with complete mesocolic excision (CME) [9], often stemming from vascular anatomy variations [40, 41]. It is one of the more common sources of bleeding in this procedure, alongside injury to the ileocolic artery stump or superior mesenteric vein [39, 42].

The distances between the ICV-GTH, the ICA-MCA, and the number and caliber of JVs have previously been reported by our research group [27]. These distances (length and caliber), as well as the named structures, are anatomical landmarks that radiologist or any dedicated specialist can add in there reporting, thus facilitating the surgeon’s orientation when analyzing CTs [11].

Preoperative three-dimensional vascular reconstruction may improve recognition of patient-specific middle colic venous anatomy and provide a more detailed understanding of confluence patterns in the D3 region. However, this descriptive study does not determine whether such imaging alters operative strategy, reduces vascular injury, or improves oncologic outcomes. Any selective vascular control strategy based on these findings should therefore be considered hypothetical and requires prospective clinical validation, as described by Sanchez et al. [31].

The strength of this study lies in the robust sample size of high-quality CT scans (slice 0.67 mm). Furthermore, this technique of 3D reconstruction has been validated (NTM) at surgery [7]. Limitations of this study could be that all vascular reconstructions and anatomical classifications were performed by a single experienced clinical anatomist (BVS) who has over 1000 cases of successful manual segmentations of abdominal CT datasets in the context of clinical studies, all validated at surgery and published [24, 27]. This may introduce observer bias and limit the assessment of interobserver variability in interpreting venous anatomy and 3D‑CT reconstructions. Future studies should incorporate multiple independent observers or formal interobserver agreement analysis to strengthen the robustness and generalizability of the imaging‑based findings. A possible weakness is that traditionally small-caliber vessels (such as the PDV) are not easily visualized on standard preoperative portovenous-phase CT images.

## Conclusion

Despite a consistent presence, the MCV anatomy is profoundly complex and unpredictable due to multiplicity, alternative confluence pattern, and topographic relations to the MCA. Preoperative CT 3D vascular reconstruction may help surgeons visualize individual MCV anatomy and better intervascular visualization before right colectomy. Its impact on intraoperative decision-making, vascular injury, and oncological outcomes remains partially unknown and should be evaluated further in prospective studies.

## Supplementary Information

Below is the link to the electronic supplementary material.

## Data Availability

Data can be obtained upon request.

## References

[1] Alsabilah J, Kim WR, Kim NK (2017) Vascular Structures of the Right Colon: Incidence and Variations with Their Clinical Implications. Scand J Surg 106(2):107–115. 10.1177/1457496916650999 27215222

[2] Anania G, Campagnaro A, Chiozza M, Randolph J, Resta G, Marino S, Pedon S, Agrusa A, Cuccurullo D, Cirocchi R (2024) SICE CoDIG (ColonDx Italian Group). A SICE (Società Italiana di Chirurgia Endoscopica e Nuove Tecnologie) observational prospective multicenter study on anatomical variants of the superior mesenteric artery: intraoperative analysis during laparoscopic right hemicolectomy-CoDIG 2 database (ColonDx Italian Group). Updates Surg 76(3):933–941. 10.1007/s13304-024-01787-6 38526696 PMC11129964

[3] Andersen BT, Kazaryan AM, Stimec BV, Edwin B, Rancinger P, Ignjatovic D (2022) Personalized surgery for the splenic flexure cancer: new frontiers. Br J Surg 109(9):880–881. 10.1093/bjs/znac153 35640281 PMC10364751

[4] Andersen BT, Stimec BV, Edwin B, Kazaryan AM, Maziarz PJ, Ignjatovic D (2022) Re-interpreting mesenteric vascular anatomy on 3D virtual and/or physical models: positioning the middle colic artery bifurcation and its relevance to surgeons operating colon cancer. Surg Endosc 36(1):100–108. 10.1007/s00464-020-08242-8 33492511 PMC8741724

[5] Andersen BT, Stimec BV, Kazaryan AM, Rancinger P, Edwin B, Ignjatovic D (2022) Re-interpreting mesenteric vascular anatomy on 3D virtual and/or physical models, part II: anatomy of relevance to surgeons operating splenic flexure cancer. Surg Endosc 36(12):9136–9145. 10.1007/s00464-022-09394-5 35773607 PMC9652173

[6] Asensio JA, Petrone P, Garcia-Nuñez L, Healy M, Martin M, Kuncir E (2007) Superior mesenteric venous injuries: to ligate or to repair remains the question. J Trauma 62(3):668–675. 10.1097/01.ta.0000210434.56274.7f 17414345

[7] Banipal GS, Stimec BV, Andersen SN, Edwin B, Nesgaard JM, Šaltytė Benth J, Ignjatovic D (2024) RCC study group. Are Metastatic Central Lymph Nodes (D3 volume) in right-sided Colon Cancer a Sign of Systemic Disease? A sub-group Analysis of an Ongoing Multicenter Trial. Ann Surg 279(4):648–656. 37753647 10.1097/SLA.0000000000006099 PMC10922660

[8] Bertani E, Zugni F, Radice D, Spada F, Bonomo G, Fumagalli Romario U, Fazio N, Funicelli L (2022) Predicting resectability of primary tumor and mesenteric lymph-node masses in patients with small-intestine neuroendocrine tumors. Updates Surg 74(5):1697–1704. 10.1007/s13304-022-01251-3 35224681

[9] Croner RS, Ptok H, Merkel S, Hohenberger W (2018) Implementing complete mesocolic excision for colon cancer - mission completed? Innov Surg Sci 3(1):17–29. 10.1515/iss-2017-0042 31579762 PMC6754049

[10] Dighe S, Purkayastha S, Swift I, Tekkis PP, Darzi A, A’Hern R, Brown G (2010) Diagnostic precision of CT in local staging of colon cancers: a meta-analysis. Clin Radiol 65(9):708–719. 10.1016/j.crad.2010.01.024 20696298

[11] Flor N, Campari A, Ravelli A, Lombardi MA, Pisani Ceretti A, Maroni N, Opocher E, Cornalba G (2015) Vascular Map Combined with CT Colonography for Evaluating Candidates for Laparoscopic Colorectal Surgery. Korean J Radiol 16(4):821–826. 10.3348/kjr.2015.16.4.821 26175581 PMC4499546

[12] Gao Y, Lu Y (2018) Variations of Gastrocolic Trunk of Henle and Its Significance in Gastrocolic Surgery. Gastroenterol Res Pract 2018:3573680. 10.1155/2018/3573680 29977286 PMC6011069

[13] Gaupset R, Nesgaard JM, Kazaryan AM, Stimec BV, Edwin B, Ignjatovic D (2018) Introducing Anatomically Correct CT-Guided Laparoscopic Right Colectomy with D3 Anterior Posterior Extended Mesenterectomy: Initial Experience and Technical Pitfalls. J Laparoendosc Adv Surg Tech A 28(10):1174–1182. 10.1089/lap.2018.0059 29741975

[14] Gillot C, Hureau J, Aaron C, Martini R, Thaler G, Michels Na (1964) The Superior Mesenteric Vein, An Anatomic and Surgical Study of Eighty-One Subjects. J Int Coll Surg 41:339–369 Pmid: 14147012 14147012

[15] Hamabe A, Park S, Morita S et al (2018) Analysis of the Vascular Interrelationships Among the First Jejunal Vein, the Superior Mesenteric Artery, and the Middle Colic Artery. Ann Surg Oncol 25:1661–1667. 10.1245/s10434-018-6456-z 29616421

[16] Hohenberger W, Weber K, Matzel K, Papadopoulos T, Merkel S (2009) Standardized surgery for colonic cancer: complete mesocolic excision and central ligation–technical notes and outcome. Colorectal Dis 11(4):354–364 discussion 364-5. 10.1111/j.1463-1318.2008.01735.x 19016817

[17] Ielpo B, d’Addetta MV, Anselmo A, Rosso E, de Blasi V, Sanchez-Velazquez P, Vellalta G, Podda M, Burdio F (2024) Levels of Robotic Mesopancreas Dissection According to Malignancy and Vascular Anatomy: What Surgeons Need to Know. Ann Surg Oncol 31(3):1916–1918. 10.1245/s10434-023-14686-8 38071705 PMC10838235

[18] Ignjatovic D, Bergamaschi R, Stimec BV (2025) The forgotten nodes: a narrative review. Ann Laparosc Endosc Surg 10:6. 10.21037/ales-24-23

[19] Ignjatovic D, Stimec B, Finjord T, Bergamaschi R (2004) Venous anatomy of the right colon: three-dimensional topographic mapping of the gastrocolic trunk of Henle. Tech Coloproctol 8(1):19–21 discussion 21 – 2. 10.1007/s10151-004-0045-9 15057584

[20] Luzon JA, Kumar RP, Stimec BV, Elle OJ, Bakka AO, Edwin B, Ignjatovic D (2020) Semi-automated vs. manual 3D reconstruction of central mesenteric vascular models: the surgeon’s verdict. Surg Endosc 34(11):4890–4900. 10.1007/s00464-019-07275-y 31745632

[21] Mai PT, Hoang VT, Nguyen TTT, Vo THT, Le VP (2025) Portal vein confluence variants on contrast-enhanced CT: distribution, IMV-confluence distance, and novel patterns in 199 adults. Surg Radiol Anat 47(1):237. 10.1007/s00276-025-03754-1 41120551

[22] Maki Y, Mizutani M, Morimoto M, Kawai T, Nakagawa M, Ozawa Y, Takeuchi M, Maki H, Kurosaka K, Shibamoto Y (2016) The variations of the middle colic vein tributaries: depiction by three-dimensional CT angiography. Br J Radiol 89(1063):20150841. 10.1259/bjr.20150841 27109734 PMC5257301

[23] Mason JD, Khan JS, Ahmed S, Coyne P, Read JW, Bundred J, Buczacki SJA, Harikrishnan A, Cunningham C (2026) Association of Coloproctology of Great Britain & Ireland (ACPGBI). ACPGBI position statement on the role of standard high-quality right hemicolectomy and complete mesocolic excision in right-sided colon cancer. Colorectal Dis 28(5):e70495. 10.1111/codi.70495 42175521

[24] Moen TM, Stimec B, Ignjatovic D (2022) The Use of a Vascular Roadmap at Surgery Evens out Surgeons’ Expectations on Operating Time, Blood Loss, Lymph Nodes Harvest and Operative Difficulty when Performing Right Colectomy with Extended D3 Mesenterectomy. Chirurgia (Romania) 117(5):579–584. 10.21614/chirurgia.2766 36318688

[25] Negoi I, Beuran M, Hostiuc S, Negoi RI, Inoue Y (2018) Surgical Anatomy of the Superior Mesenteric Vessels Related to Colon and Pancreatic Surgery: A Systematic Review and Meta-Analysis. Sci Rep 8(1):4184. 10.1038/s41598-018-22641-x 29520096 PMC5843657

[26] Nepal P, Mori S, Kita Y, Tanabe K, Baba K, Sasaki K, Kurahara H, Arigami T, Ohtsuka T (2021) Anatomical study of the inferior mesenteric vein using three-dimensional computed tomography angiography in laparoscopy-assisted surgery for left-sided colorectal cancer. Surg Today 51(10):1665–1670. 10.1007/s00595-021-02292-8 33893527

[27] Nerad E, Lahaye MJ, Maas M, Nelemans P, Bakers FC, Beets GL, Beets-Tan RG (2016) Diagnostic Accuracy of CT for Local Staging of Colon Cancer: A Systematic Review and Meta-Analysis. AJR Am J Roentgenol 207(5):984–995. 10.2214/AJR.15.15785 27490941

[28] Nesgaard JM, Stimec BV, Bakka AO, Edwin B, Ignjatovic D (2015) RCC study group. Navigating the mesentery: a comparative pre- and per-operative visualization of the vascular anatomy. Colorectal Dis 17(9):810–818. 10.1111/codi.13003 25988347

[29] Nigah S, Patra A, Chumber S (2022) Analysis of the Variations in the Colic Branching Pattern of the Superior Mesenteric Artery: A Cadaveric Study With Proposal to Modify Its Current Anatomical Classification. Cureus 14(5):e25025. 10.7759/cureus.25025 35719766 PMC9199573

[30] Ogino T, Takemasa I, Horitsugi G, Furuyashiki M, Ohta K, Uemura M, Nishimura J, Hata T, Mizushima T, Yamamoto H, Doki Y, Mori M (2014) Preoperative evaluation of venous anatomy in laparoscopic complete mesocolic excision for right colon cancer. Ann Surg Oncol 21(Suppl 3):S429–S435. 10.1245/s10434-014-3572-2 24633663

[31] Sánchez-Rodríguez M, Pastor C, Jiménez-Ruiz J, Dujovne-Lindenbaum P, Craus-Miguel A, Tejedor P (2026) D3 Lymph Node Dissection in Right-Sided Colon Cancer: A Video Insight into Key Vascular Variations. Dis Colon Rectum. 10.1097/DCR.0000000000004128 41733135

[32] Spasojevic M, Stimec BV, Fasel JF, Terraz S, Ignjatovic D (2011) 3D relations between right colon arteries and the superior mesenteric vein: a preliminary study with multidetector computed tomography. Surg Endosc 25(6):1883–1886. 10.1007/s00464-010-1480-5 21136104

[33] Standring S (ed) (2021) Gray’s Anatomy: The Anatomical Basis of Clinical Practice, 42nd edn. Elsevier Limited, New York

[34] Stefura T, Kacprzyk A, Droś J, Pędziwiatr M, Major P, Hołda MK (2018) The venous trunk of henle (gastrocolic trunk): A systematic review and meta-analysis of its prevalence, dimensions, and tributary variations. Clin Anat 31(8):1109–1121. 10.1002/ca.23228 30133829

[35] Stelzner S, Hohenberger W, Weber K, West NP, Witzigmann H, Wedel T (2016) Anatomy of the transverse colon revisited with respect to complete mesocolic excision and possible pathways of aberrant lymphatic tumor spread. Int J Colorectal Dis 31(2):377–384. 10.1007/s00384-015-2434-0 26546443

[36] Stimec BV, Ignjatovic D (2020) Navigating the mesentery: Part III. Unusual anatomy of ileocolic vessels. Colorectal Dis 22(12):1949–1957. 10.1111/codi.15284 32734680

[37] Sun KK, Zhao H (2020) Vascular anatomical variation in laparoscopic right hemicolectomy. Asian J Surg 43(1):9–12. 10.1016/j.asjsur.2019.03.013 30979567

[38] Willard CD, Kjaestad E, Stimec BV et al (2019) Preoperative anatomical road mapping reduces variability of operating time, estimated blood loss, and lymph node yield in right colectomy with extended D3 mesenterectomy for cancer. Int J Colorectal Dis 34(1):151–160. 10.1007/s00384-018-3177-5 30386889

[39] Wu C, Ye K, Wu Y, Chen Q, Xu J, Lin J, Kang W (2019) Variations in right colic vascular anatomy observed during laparoscopic right colectomy. World J Surg Oncol 17(1):16. 10.1186/s12957-019-1561-4 30636641 PMC6330569

[40] Yamaguchi S, Kuroyanagi H, Milsom JW, Sim R, Shimada H (2002) Venous anatomy of the right colon: precise structure of the major veins and gastrocolic trunk in 58 cadavers. Dis Colon Rectum 45(10):1337–1340. 10.1097/01.DCR.0000027284.76452.84 12394432

[41] Ye K, Lin J, Sun Y, Wu Y, Xu J, He S (2018) Variation and treatment of vessels in laparoscopic right hemicolectomy. Surg Endosc 32(3):1583–1584. 10.1007/s00464-017-5751-2 28733739

[42] Zhao LY, Li GX, Zhang C, Yu J, Deng HJ, Wang YN, Hu YF, Cheng X (2012) [Vascular anatomy of the right colon and vascular complications during laparoscopic surgery]. Zhonghua Wei Chang Wai Ke Za Zhi 15(4):336–341 22539376

